# Stress, Trauma, and Resilience Among U.S. Asian Older Adults: Findings From the Rutgers Asian RCMAR

**DOI:** 10.1093/geroni/igaa057.2124

**Published:** 2020-12-16

**Authors:** XinQi Dong, Melissa Simon, Bei Wu

## Abstract

U.S. Asians are the fastest growing group of older adults in the nation, increasing by 68% from 2000-2018. However, research on the psychological wellbeing of this population is limited. Drawing on the research of Rutgers Asian RCMAR Scientists, this symposium will address the impacts of stress, trauma and resilience on the psychological wellbeing of diverse groups of U.S. Asian older adults. Session 1 will assess the prevalence of psychological distress among older LGBT and non-LGBT U.S. Asian older adults, and the role of discrimination in medical care and intimate violence on psychological distress. Session 2 will take a mixed-methods approach to examining caregiver burden and depressive symptoms of Chinese American spouses and adult-children who provided care for their spouse or parents with dementia. Session 3 will explore the risk and protective factors for the mental health of sexual minority U.S. Asian older adults using data from the Research Program on Genes, Environment and Health. Session 4 will identify different patterns of coping repertoires of older immigrants, based on a combination of individual, family, and community coping resources, and the optimal coping repertoire that is associated with the best psychological outcomes. In summation, this symposium describes the psychological wellbeing of diverse groups of U.S. Asian older adults, including sexual minority, caregiver and immigrant groups. The symposium addresses both risk factors and the protective factors and coping mechanisms that mediate and mitigate psychological wellbeing and aims to inform interventions to improve psychological wellbeing outcomes in U.S. Asian older adults.

